# A new approach to alkaloid-like systems: synthesis and crystal structure of 1-(2-acetyl-11-meth­oxy-5,6-di­hydro­[1,3]dioxolo[4,5-*g*]pyrrolo­[2,1-*a*]isoquinolin-1-yl)propan-2-one

**DOI:** 10.1107/S2056989017015110

**Published:** 2017-10-20

**Authors:** Le Tuan Anh, Alexander A. Titov, Reza Samavati, Leonid G. Voskressensky, Alexey V. Varlamov, Victor N. Khrustalev

**Affiliations:** a Department of Pharmaceutical Chemistry, Faculty of Chemistry, VNU University of Science, 19 Le Thanh Tong, Hoan Kiem, Hanoi 100000, Vietnam; b Organic Chemistry Department, Peoples’ Friendship University of Russia, (RUDN University), Miklukho-Maklay St., 6, Moscow 117198, Russian Federation; c Inorganic Chemistry Department, Peoples’ Friendship University of Russia, (RUDN University), Miklukho-Maklay St., 6, Moscow 117198, Russian Federation

**Keywords:** alkaloids, lamellarin, cotarnine, di­hydro­pyrrolo­[2,1-*a*]iso­quinolines, domino reaction, crystal structure

## Abstract

The title compound, C_19_H_19_NO_5_, (I), is the product of a domino reaction between cotarnine chloride and acetyl­acetylene catalysed by copper(I) iodide. The mol­ecule of (I) comprises a fused tetra­cyclic system containing two terminal five-membered rings (pyrrole and 1,3-dioxole) and two central six-membered rings (di­hydro­pyridine and benzene). The five-membered 1,3-dioxole ring has an envelope conformation and the central six-membered di­hydro­pyridine ring adopts a twist-boat conformation. The acyl substituent is almost coplanar with the pyrrole ring, whereas the meth­oxy substituent is twisted by 27.93 (16)° relative to the benzene ring. The 2-oxopropan-1-yl substituent is roughly perpendicular to the pyrrole ring. In the crystal, mol­ecules are stacked along the *a*-axis direction; the stacks are linked by weak C—H⋯O hydrogen bonds into puckered layers lying parallel to (001).

## Abstract 2

1-(2-Acetyl-11-meth­oxy-5,6-di­hydro­[1,3]dioxolo[4,5-*g*]pyrrolo­[2,1-*a*]isoquinolin-1-yl)propan-2-one, the product of a domino reaction between cotarnine chloride and acetyl­acetylene catalysed by copper(I) iodide, was studied by X-ray diffraction.

## Chemical context   

The 5,6-di­hydro­pyrrolo­[2,1-*a*]iso­quinoline fragment is included in several natural products, for example in lamellarin I and K alkaloids, which possess a variety of biological properties, in particular, anti­tumor activity (Komatsubara *et al.*, 2014 ▸; Imperatore *et al.*, 2014 ▸).

The di­hydro­pyrrolo­[2,1-*a*]iso­quinoline skeleton can be constructed in two different ways. The first way is annelation of a pyrrole ring to an isoquiniline fragment (Ma *et al.*, 2014 ▸; Fujiya *et al.*, 2016 ▸; Nekkanti *et al.*, 2016 ▸). The second one, in contrast, is annelation of an isoquiniline fragment to pyrrole derivatives (Sun *et al.*, 2012 ▸; Wiest *et al.*, 2016 ▸). Previously, we developed synthetic approaches to substituted pyrrolo­[2,1-*a*]iso­quinolines *via* the inter­action of 1-aroyl-3,4-di­hydro­isoquinilines or 1-ethynyl-1,2,3,4-tetra­hydro­iso­quinolines with activated alkynes (Voskressensky, Titov *et al.*, 2016 ▸; Voskressensky *et al.*, 2017 ▸).

It is of fundamental importance for the preparation of 2,3-bifunctional substituted pyrrolo­[2,1-*a*]iso­quinolines to study the inter­action of iminium salts with activated alkynes. In this work, we modified the approach to the synthesis of alkaloid-like compounds by the reaction of cotarnine chloride with activated alkynes in the presence of copper halogenides as a catalyst. The synthetic method proposed is new and original. This process includes the formation of the pyrrole ring and its functionalization, which is necessary for the chemical diversity of pyrrolo­iso­quinoline systems.

The title compound (I) is a product of a new domino reaction between cotarnine chloride and acetyl­acetylene catalysed by copper(I) iodide. The reaction sequence starts with nucleophilic addition of copper(I) acetyl­ide to cotarnine chloride followed by [2,3]-cyclo­addition and aromatization of the pyrrole ring (Voskressensky, Borisova *et al.*, 2016 ▸). The main speciality of the reaction is the conversion of the acetyl­ethynyl fragment to acetyl­methyl when the pyrrole ring is formed in an aprotic solvent. The structure of the product (I) was unambiguously established by an X-ray diffraction study.
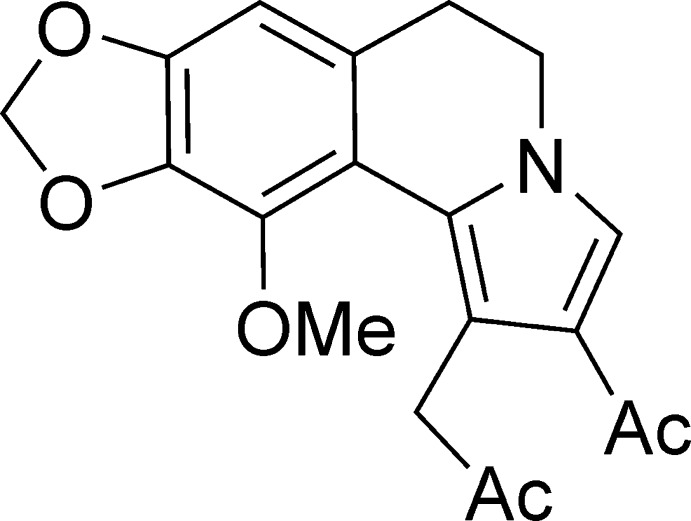


## Structural commentary   

**Figure 1:**
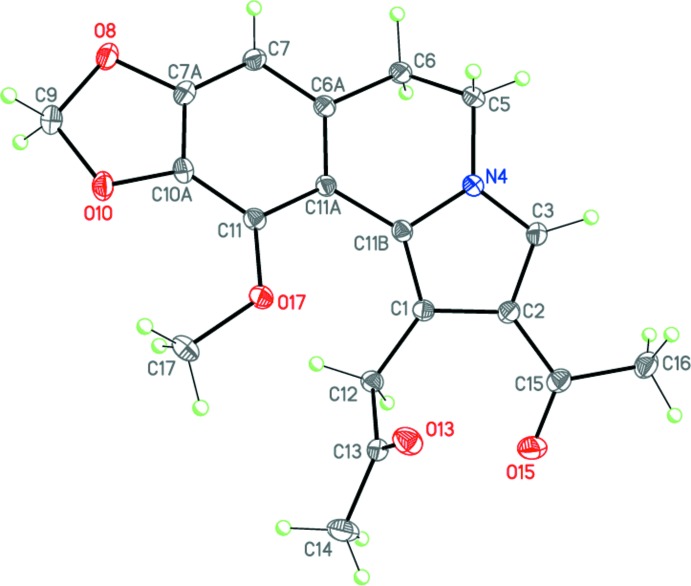
Mol­ecular structure of (I). Displacement ellipsoids are drawn at the 50% probability level. H atoms are represented as small spheres of arbitrary radius.

The mol­ecule of (I), representing a new alkaloid-like skeleton, comprises a fused tetra­cyclic system containing two terminal five-membered rings (pyrrole and 1,3-dioxole) and two central six-membered rings (di­hydro­pyridine and benzene) (Fig. 1 ▸). The five-membered 1,3-dioxole ring has its usual shallow envelope conformation, with the methyl­ene group as the flap, and the central six-membered di­hydro­pyridine ring adopts a twist-boat conformation. The dihedral angle between the pyrrole and benzene rings is 29.69 (3)°. The nitro­gen N4 atom is essentially planar (sum of bond angles = 359.73°). The acyl substituent is almost coplanar with the pyrrole ring (r.m.s. deviation for non-hydrogen atoms = 0.012 Å), whereas the meth­oxy substituent is twisted by 27.93 (16)° relative to the benzene ring. The propan-2-one-1-yl substituent is roughly perpendicular to the pyrrole ring, the dihedral angle being 76.81 (5)°, because of steric reasons.

## Supra­molecular features   

**Figure 2:**
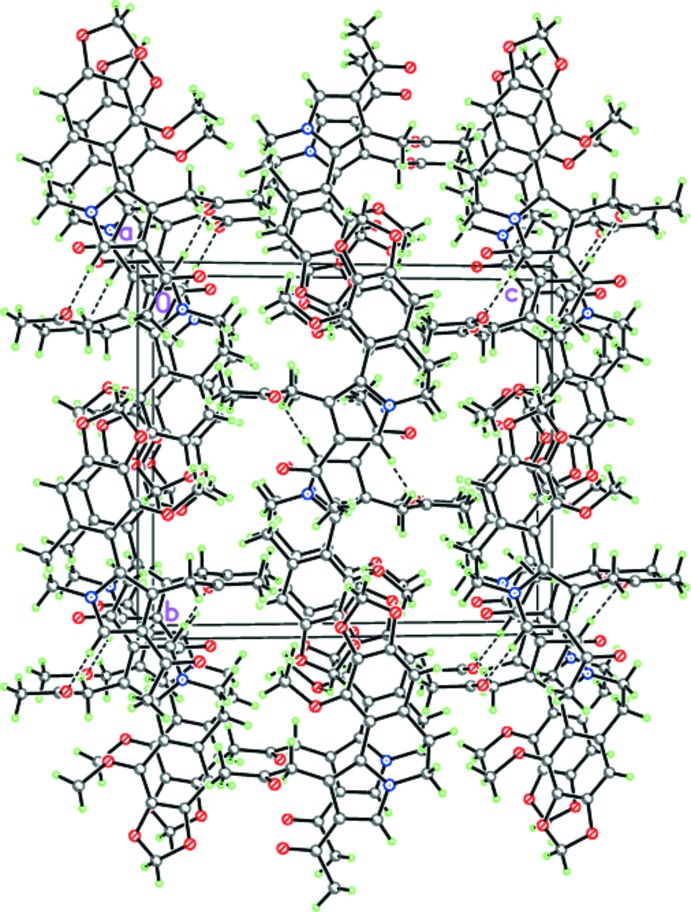
Crystal structure of (I) illustrating the hydrogen-bonded layers parallel to (001). Dashed lines indicate the inter­molecular C—H⋯O hydrogen bonds.

The crystal packing of mol­ecules of (I) involves stacking along the *a-*axis direction (Fig. 2 ▸), with mol­ecules linked by weak C—H⋯O hydrogen bonds into puckered layers lying parallel to (001) (Table 1 ▸, Fig. 2 ▸).

## Synthesis and crystallization   

**Figure 3:**
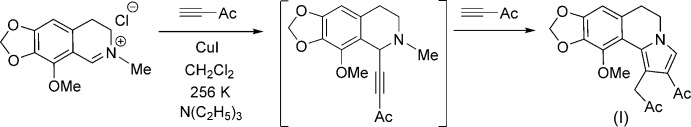
Synthesis of (I) using a domino reaction between cotarnine chloride and acetyl­acetylene catalysed by copper(I) iodide.

Acetyl­acetylene (0.27 g, 3.9 mmol) was added to a stirred suspension of cotarnine chloride (0.10 g, 0.39 mmol) and CuI (0.011 g, 0.059 mmol) in CH_2_Cl_2_ (10 ml) under Ar at 256 K (Fig. 3 ▸). After stirring at 256 K for 1 h, tri­ethyl­amine (0.059 g, 0.59 mmol) was added to the mixture under Ar at 256 K. The reaction mixture was stirred at 256 K for 30 min, and brought to room temperature and stirred for three days. The reaction progress was monitored by TLC (eluent EtOH). After the completion, the solvent was removed in vacuum, and the residue separated by column chromatography on silica gel (EtOAc–hexane, 1:1). After removing the solvent, the residue was recrystallized from an EtOAc–hexane solvent mixture to give 37 mg (28% yield) of yellow–orange crystals of the title compound, m.p. = 448–450 K (EtOAc–hexa­ne).

^1^H NMR (CDCl_3_, 600 MHz): *δ* = 2.29 (3*H*, *s*, COCH_3_); 2.38 (3H, *s*, COCH_3_); 2.85–2.87 (2H, *m*, 6-CH_2_); 3.80 (3H, *s*, 11-OCH_3_); 3.94–3.96 (2H, *m*, 5-CH_2_); 4.00 (2H, *s*, CH_2_COCH_3_); 5.96 (2H, *s*, 9-CH_2_); 6.49 (1H, *s*, H-7); 7.37 (1H, *s*, H-3); ^13^C NMR (CDCl_3_, 150 MHz): *δ* = 27.4, 29.5, 31.5, 42.4, 44.9, 59.9, 101.0, 103.3, 115.4, 115.8, 123.0, 126.5, 127.5, 129.5, 136.5, 139.9, 147.8, 193.1, 207.4; *m*/*z*: 341 [*M*]^+^ (67), 299 (33), 298 (100), 284 (18), 283 (72), 282 (54), 268 (5), 256 (31), 255 (28), 254 (21), 241 (15), 240 (47), 212 (6), 182 (5), 168 (7), 167 (7), 154 (12), 127 (7), 43 (16). Analysis calculated for C_19_H_19_NO_5_ (%): C 66.85, H 5.61, N 4.10; found (%): C 66.92, H 5.55, N 4.15.

## Refinement   

Crystal data, data collection and structure refinement details are summarized in Table 2 ▸. Hydrogen atoms were placed in calculated positions with C—H = 0.95–0.99 Å and refined using the riding model with fixed isotropic displacement parameters [*U*_iso_(H) = 1.5*U*_eq_(C) for the CH_3_-groups and 1.2*U*_eq_(C) for the other groups].

## Supplementary Material

Crystal structure: contains datablock(s) global, I. DOI: 10.1107/S2056989017015110/hb7707sup1.cif

Structure factors: contains datablock(s) I. DOI: 10.1107/S2056989017015110/hb7707Isup2.hkl

CCDC reference: 1580424

Additional supporting information:  crystallographic information; 3D view; checkCIF report

## Figures and Tables

**Table 1:** Hydrogen-bond geometry (Å, °)

*D*—H⋯*A*	*D*—H	H⋯*A*	*D*⋯*A*	*D*—H⋯*A*
C3—H3⋯O13^i^	0.95	2.44	3.2840 (14)	147
C9—H9*A*⋯O17^ii^	0.99	2.46	3.2207 (15)	133

Symmetry codes: (i) 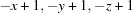; (ii) 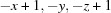.

**Table 2:** Experimental details

Crystal data	
Chemical formula	C_19_H_19_NO_5_
*M*_r_	341.35
Crystal system, space group	Monoclinic, *P*2_1_/*n*
Temperature (K)	120
*a*, *b*, *c* (Å)	7.2782 (4), 14.0016 (7), 15.7852 (8)
β (°)	99.546 (1)
*V* (Å^3^)	1586.34 (14)
*Z*	4
Radiation type	Mo *K*α
μ (mm^−1^)	0.10
Crystal size (mm)	0.20 × 0.15 × 0.15

Data collection	
Diffractometer	Bruker APEXII CCD
Absorption correction	Multi-scan (*SADABS*; Sheldrick, 2003 ▸)
*T*_min_, *T*_max_	0.970, 0.980
No. of measured, independent and observed [*I* > 2σ(*I*)] reflections	24316, 5762, 4535
*R*_int_	0.042
(sin θ/λ)_max_ (Å^−1^)	0.758

Refinement	
*R*[*F* ^2^ > 2σ(*F* ^2^)], *wR*(*F* ^2^), *S*	0.048, 0.133, 1.03
No. of reflections	5762
No. of parameters	229
H-atom treatment	H-atom parameters constrained
Δρ_max_, Δρ_min_ (e Å^−3^)	0.46, −0.38

Computer programs: *APEX2* (Bruker, 2005 ▸), *SAINT* (Bruker, 2001 ▸), *SHELXT* (Sheldrick, 2015*a* ▸), *SHELXL2014* (Sheldrick, 2015*b* ▸) and *SHELXTL* (Sheldrick, 2008 ▸).

## supplementary crystallographic information

### Crystal data

**Table:**

C_19_H_19_NO_5_	*F*(000) = 720
*M**_r_* = 341.35	*D*_x_ = 1.429 Mg m^−^^3^
Monoclinic, *P*2_1_/*n*	Mo *K*α radiation, λ = 0.71073 Å
*a* = 7.2782 (4) Å	Cell parameters from 5328 reflections
*b* = 14.0016 (7) Å	θ = 2.6–31.9°
*c* = 15.7852 (8) Å	µ = 0.10 mm^−^^1^
β = 99.546 (1)°	*T* = 120 K
*V* = 1586.34 (14) Å^3^	Prism, orange
*Z* = 4	0.20 × 0.15 × 0.15 mm

### Data collection

**Table:**

Bruker APEXII CCD diffractometer	4535 reflections with *I* > 2σ(*I*)
Radiation source: fine-focus seales tube	*R*_int_ = 0.042
φ and ω scans	θ_max_ = 32.6°, θ_min_ = 2.0°
Absorption correction: multi-scan (SADABS; Sheldrick, 2003)	*h* = −11→10
*T*_min_ = 0.970, *T*_max_ = 0.980	*k* = −20→21
24316 measured reflections	*l* = −23→23
5762 independent reflections	

### Refinement

**Table:**

Refinement on *F*^2^	Primary atom site location: difference Fourier map
Least-squares matrix: full	Secondary atom site location: difference Fourier map
*R*[*F*^2^ > 2σ(*F*^2^)] = 0.048	Hydrogen site location: inferred from neighbouring sites
*wR*(*F*^2^) = 0.133	H-atom parameters constrained
*S* = 1.03	*w* = 1/[σ^2^(*F*_o_^2^) + (0.068*P*)^2^ + 0.4867*P*] where *P* = (*F*_o_^2^ + 2*F*_c_^2^)/3
5762 reflections	(Δ/σ)_max_ < 0.001
229 parameters	Δρ_max_ = 0.46 e Å^−^^3^
0 restraints	Δρ_min_ = −0.38 e Å^−^^3^

### Special details

**Table:**

Geometry. All esds (except the esd in the dihedral angle between two l.s. planes) are estimated using the full covariance matrix. The cell esds are taken into account individually in the estimation of esds in distances, angles and torsion angles; correlations between esds in cell parameters are only used when they are defined by crystal symmetry. An approximate (isotropic) treatment of cell esds is used for estimating esds involving l.s. planes.

### Fractional atomic coordinates and isotropic or equivalent isotropic displacement parameters (Å^2^)

**Table:**

	*x*	*y*	*z*	*U*_iso_*/*U*_eq_	
C1	0.20836 (15)	0.37136 (8)	0.45572 (7)	0.01256 (19)	
C2	0.24234 (15)	0.46920 (8)	0.48054 (7)	0.0138 (2)	
C3	0.28874 (16)	0.47143 (8)	0.56943 (7)	0.0147 (2)	
H3	0.3177	0.5269	0.6036	0.018*	
N4	0.28561 (14)	0.38096 (7)	0.59883 (6)	0.01391 (18)	
C5	0.30680 (18)	0.35126 (8)	0.68855 (7)	0.0174 (2)	
H5A	0.2856	0.4062	0.7252	0.021*	
H5B	0.4344	0.3268	0.7081	0.021*	
C6	0.16472 (17)	0.27329 (8)	0.69533 (7)	0.0165 (2)	
H6A	0.1820	0.2487	0.7549	0.020*	
H6B	0.0373	0.2999	0.6812	0.020*	
C6A	0.18777 (15)	0.19242 (8)	0.63407 (7)	0.01320 (19)	
C7	0.17044 (16)	0.09736 (8)	0.65958 (7)	0.0150 (2)	
H7	0.1389	0.0818	0.7140	0.018*	
C7A	0.20129 (16)	0.02752 (8)	0.60209 (7)	0.0147 (2)	
O8	0.19904 (13)	−0.06993 (6)	0.61417 (6)	0.01939 (18)	
C9	0.22878 (17)	−0.11078 (8)	0.53375 (8)	0.0169 (2)	
H9A	0.3207	−0.1634	0.5441	0.020*	
H9B	0.1105	−0.1367	0.5020	0.020*	
O10	0.29712 (13)	−0.03606 (6)	0.48500 (6)	0.01822 (18)	
C10A	0.25597 (15)	0.04791 (8)	0.52391 (7)	0.0137 (2)	
C11	0.27333 (15)	0.14145 (8)	0.49713 (7)	0.01233 (19)	
C11A	0.23175 (15)	0.21532 (8)	0.55265 (7)	0.01190 (19)	
C11B	0.24022 (15)	0.31718 (8)	0.53044 (7)	0.01223 (19)	
C12	0.13624 (15)	0.34137 (8)	0.36538 (7)	0.0141 (2)	
H12A	0.0251	0.3802	0.3431	0.017*	
H12B	0.0963	0.2738	0.3656	0.017*	
C13	0.27738 (16)	0.35160 (8)	0.30483 (7)	0.0145 (2)	
O13	0.44297 (12)	0.36125 (7)	0.33022 (6)	0.02009 (19)	
C14	0.19984 (19)	0.34580 (11)	0.21036 (8)	0.0235 (3)	
H14A	0.2926	0.3697	0.1771	0.035*	
H14B	0.0867	0.3848	0.1979	0.035*	
H14C	0.1698	0.2792	0.1946	0.035*	
C15	0.23042 (17)	0.55343 (8)	0.42517 (8)	0.0165 (2)	
O15	0.19111 (15)	0.54807 (7)	0.34639 (6)	0.0253 (2)	
C16	0.26736 (18)	0.64920 (8)	0.46885 (8)	0.0196 (2)	
H16A	0.2745	0.6986	0.4255	0.029*	
H16B	0.3855	0.6467	0.5089	0.029*	
H16C	0.1660	0.6644	0.5005	0.029*	
O17	0.33691 (12)	0.16770 (6)	0.42396 (5)	0.01608 (17)	
C17	0.31065 (19)	0.10236 (9)	0.35248 (8)	0.0206 (2)	
H17A	0.3391	0.1348	0.3012	0.031*	
H17B	0.1811	0.0803	0.3418	0.031*	
H17C	0.3939	0.0474	0.3658	0.031*	

### Atomic displacement parameters (Å^2^)

**Table:**

	*U*^11^	*U*^22^	*U*^33^	*U*^12^	*U*^13^	*U*^23^
C1	0.0131 (4)	0.0129 (4)	0.0122 (4)	−0.0003 (4)	0.0034 (4)	−0.0007 (4)
C2	0.0154 (5)	0.0121 (5)	0.0143 (5)	0.0005 (4)	0.0036 (4)	0.0009 (4)
C3	0.0185 (5)	0.0114 (4)	0.0146 (5)	−0.0013 (4)	0.0035 (4)	−0.0010 (4)
N4	0.0184 (4)	0.0119 (4)	0.0113 (4)	−0.0009 (3)	0.0020 (3)	−0.0008 (3)
C5	0.0259 (6)	0.0148 (5)	0.0108 (4)	−0.0005 (4)	0.0007 (4)	−0.0013 (4)
C6	0.0242 (6)	0.0138 (5)	0.0126 (5)	0.0014 (4)	0.0064 (4)	−0.0008 (4)
C6A	0.0148 (5)	0.0129 (4)	0.0124 (4)	0.0006 (4)	0.0034 (4)	−0.0003 (4)
C7	0.0188 (5)	0.0133 (5)	0.0135 (5)	0.0010 (4)	0.0046 (4)	0.0016 (4)
C7A	0.0156 (5)	0.0117 (4)	0.0167 (5)	0.0002 (4)	0.0027 (4)	0.0013 (4)
O8	0.0291 (5)	0.0110 (4)	0.0192 (4)	0.0002 (3)	0.0077 (3)	0.0005 (3)
C9	0.0178 (5)	0.0121 (5)	0.0210 (5)	−0.0008 (4)	0.0039 (4)	−0.0017 (4)
O10	0.0249 (4)	0.0111 (4)	0.0204 (4)	−0.0003 (3)	0.0088 (3)	−0.0032 (3)
C10A	0.0146 (5)	0.0117 (4)	0.0152 (5)	−0.0001 (4)	0.0038 (4)	−0.0024 (4)
C11	0.0125 (4)	0.0135 (5)	0.0117 (4)	−0.0007 (4)	0.0040 (3)	−0.0010 (4)
C11A	0.0128 (4)	0.0115 (4)	0.0115 (4)	−0.0005 (3)	0.0020 (3)	−0.0009 (3)
C11B	0.0129 (4)	0.0122 (4)	0.0119 (4)	−0.0007 (3)	0.0031 (4)	−0.0012 (3)
C12	0.0140 (5)	0.0155 (5)	0.0124 (4)	−0.0014 (4)	0.0015 (4)	−0.0002 (4)
C13	0.0189 (5)	0.0121 (4)	0.0128 (4)	−0.0014 (4)	0.0040 (4)	−0.0004 (4)
O13	0.0177 (4)	0.0258 (5)	0.0173 (4)	−0.0038 (3)	0.0045 (3)	−0.0033 (3)
C14	0.0257 (6)	0.0330 (7)	0.0120 (5)	−0.0056 (5)	0.0035 (4)	−0.0004 (5)
C15	0.0181 (5)	0.0145 (5)	0.0174 (5)	0.0015 (4)	0.0046 (4)	0.0021 (4)
O15	0.0392 (6)	0.0209 (4)	0.0156 (4)	0.0024 (4)	0.0038 (4)	0.0038 (3)
C16	0.0235 (6)	0.0131 (5)	0.0224 (6)	0.0001 (4)	0.0041 (5)	0.0021 (4)
O17	0.0217 (4)	0.0151 (4)	0.0132 (4)	−0.0011 (3)	0.0081 (3)	−0.0022 (3)
C17	0.0281 (6)	0.0210 (6)	0.0138 (5)	0.0002 (5)	0.0061 (4)	−0.0049 (4)

### Geometric parameters (Å, º)

**Table:**

C1—C11B	1.3890 (15)	C9—H9B	0.9900
C1—C2	1.4350 (15)	O10—C10A	1.3820 (13)
C1—C12	1.4957 (15)	C10A—C11	1.3884 (15)
C2—C3	1.3880 (16)	C11—O17	1.3640 (13)
C2—C15	1.4620 (16)	C11—C11A	1.4204 (15)
C3—N4	1.3505 (14)	C11A—C11B	1.4723 (15)
C3—H3	0.9500	C12—C13	1.5215 (16)
N4—C11B	1.3977 (14)	C12—H12A	0.9900
N4—C5	1.4595 (14)	C12—H12B	0.9900
C5—C6	1.5197 (17)	C13—O13	1.2129 (14)
C5—H5A	0.9900	C13—C14	1.5066 (16)
C5—H5B	0.9900	C14—H14A	0.9800
C6—C6A	1.5162 (15)	C14—H14B	0.9800
C6—H6A	0.9900	C14—H14C	0.9800
C6—H6B	0.9900	C15—O15	1.2312 (15)
C6A—C7	1.4022 (16)	C15—C16	1.5114 (17)
C6A—C11A	1.4119 (15)	C16—H16A	0.9800
C7—C7A	1.3777 (16)	C16—H16B	0.9800
C7—H7	0.9500	C16—H16C	0.9800
C7A—O8	1.3783 (14)	O17—C17	1.4406 (14)
C7A—C10A	1.3877 (16)	C17—H17A	0.9800
O8—C9	1.4411 (14)	C17—H17B	0.9800
C9—O10	1.4355 (15)	C17—H17C	0.9800
C9—H9A	0.9900		
			
C11B—C1—C2	107.01 (9)	O10—C10A—C11	129.04 (10)
C11B—C1—C12	129.72 (10)	C7A—C10A—C11	121.25 (10)
C2—C1—C12	123.07 (10)	O17—C11—C10A	124.94 (10)
C3—C2—C1	107.43 (10)	O17—C11—C11A	117.59 (9)
C3—C2—C15	124.52 (10)	C10A—C11—C11A	117.37 (10)
C1—C2—C15	128.05 (10)	C6A—C11A—C11	119.99 (10)
N4—C3—C2	108.12 (10)	C6A—C11A—C11B	117.49 (9)
N4—C3—H3	125.9	C11—C11A—C11B	122.47 (10)
C2—C3—H3	125.9	C1—C11B—N4	106.96 (9)
C3—N4—C11B	110.44 (9)	C1—C11B—C11A	136.34 (10)
C3—N4—C5	126.55 (9)	N4—C11B—C11A	116.67 (9)
C11B—N4—C5	122.74 (9)	C1—C12—C13	113.94 (9)
N4—C5—C6	108.02 (9)	C1—C12—H12A	108.8
N4—C5—H5A	110.1	C13—C12—H12A	108.8
C6—C5—H5A	110.1	C1—C12—H12B	108.8
N4—C5—H5B	110.1	C13—C12—H12B	108.8
C6—C5—H5B	110.1	H12A—C12—H12B	107.7
H5A—C5—H5B	108.4	O13—C13—C14	121.44 (11)
C6A—C6—C5	110.06 (9)	O13—C13—C12	122.70 (10)
C6A—C6—H6A	109.6	C14—C13—C12	115.83 (10)
C5—C6—H6A	109.6	C13—C14—H14A	109.5
C6A—C6—H6B	109.6	C13—C14—H14B	109.5
C5—C6—H6B	109.6	H14A—C14—H14B	109.5
H6A—C6—H6B	108.2	C13—C14—H14C	109.5
C7—C6A—C11A	121.45 (10)	H14A—C14—H14C	109.5
C7—C6A—C6	120.06 (10)	H14B—C14—H14C	109.5
C11A—C6A—C6	118.46 (10)	O15—C15—C2	122.38 (11)
C7A—C7—C6A	116.89 (10)	O15—C15—C16	120.58 (11)
C7A—C7—H7	121.6	C2—C15—C16	117.03 (10)
C6A—C7—H7	121.6	C15—C16—H16A	109.5
C7—C7A—O8	127.20 (10)	C15—C16—H16B	109.5
C7—C7A—C10A	122.82 (10)	H16A—C16—H16B	109.5
O8—C7A—C10A	109.78 (10)	C15—C16—H16C	109.5
C7A—O8—C9	105.31 (9)	H16A—C16—H16C	109.5
O10—C9—O8	107.40 (9)	H16B—C16—H16C	109.5
O10—C9—H9A	110.2	C11—O17—C17	118.20 (9)
O8—C9—H9A	110.2	O17—C17—H17A	109.5
O10—C9—H9B	110.2	O17—C17—H17B	109.5
O8—C9—H9B	110.2	H17A—C17—H17B	109.5
H9A—C9—H9B	108.5	O17—C17—H17C	109.5
C10A—O10—C9	105.18 (9)	H17A—C17—H17C	109.5
O10—C10A—C7A	109.60 (10)	H17B—C17—H17C	109.5
			
C11B—C1—C2—C3	−1.55 (13)	C7—C6A—C11A—C11	−4.76 (17)
C12—C1—C2—C3	173.73 (10)	C6—C6A—C11A—C11	173.48 (10)
C11B—C1—C2—C15	179.00 (11)	C7—C6A—C11A—C11B	177.82 (10)
C12—C1—C2—C15	−5.72 (18)	C6—C6A—C11A—C11B	−3.93 (15)
C1—C2—C3—N4	0.40 (13)	O17—C11—C11A—C6A	−172.16 (10)
C15—C2—C3—N4	179.88 (11)	C10A—C11—C11A—C6A	4.35 (16)
C2—C3—N4—C11B	0.91 (13)	O17—C11—C11A—C11B	5.12 (16)
C2—C3—N4—C5	−173.22 (11)	C10A—C11—C11A—C11B	−178.37 (10)
C3—N4—C5—C6	137.61 (11)	C2—C1—C11B—N4	2.06 (12)
C11B—N4—C5—C6	−35.84 (14)	C12—C1—C11B—N4	−172.80 (11)
N4—C5—C6—C6A	55.40 (12)	C2—C1—C11B—C11A	179.75 (12)
C5—C6—C6A—C7	140.24 (11)	C12—C1—C11B—C11A	4.9 (2)
C5—C6—C6A—C11A	−38.03 (14)	C3—N4—C11B—C1	−1.89 (13)
C11A—C6A—C7—C7A	1.02 (17)	C5—N4—C11B—C1	172.50 (10)
C6—C6A—C7—C7A	−177.19 (10)	C3—N4—C11B—C11A	179.90 (10)
C6A—C7—C7A—O8	177.40 (11)	C5—N4—C11B—C11A	−5.71 (16)
C6A—C7—C7A—C10A	3.08 (17)	C6A—C11A—C11B—C1	−150.28 (13)
C7—C7A—O8—C9	176.86 (12)	C11—C11A—C11B—C1	32.37 (19)
C10A—C7A—O8—C9	−8.22 (13)	C6A—C11A—C11B—N4	27.24 (15)
C7A—O8—C9—O10	15.32 (12)	C11—C11A—C11B—N4	−150.10 (10)
O8—C9—O10—C10A	−16.59 (12)	C11B—C1—C12—C13	−114.40 (13)
C9—O10—C10A—C7A	11.69 (12)	C2—C1—C12—C13	71.46 (14)
C9—O10—C10A—C11	−172.25 (11)	C1—C12—C13—O13	16.37 (16)
C7—C7A—C10A—O10	172.97 (11)	C1—C12—C13—C14	−165.56 (10)
O8—C7A—C10A—O10	−2.22 (13)	C3—C2—C15—O15	179.01 (12)
C7—C7A—C10A—C11	−3.45 (18)	C1—C2—C15—O15	−1.6 (2)
O8—C7A—C10A—C11	−178.64 (10)	C3—C2—C15—C16	−1.43 (17)
O10—C10A—C11—O17	0.16 (19)	C1—C2—C15—C16	177.94 (11)
C7A—C10A—C11—O17	175.81 (11)	C10A—C11—O17—C17	27.93 (16)
O10—C10A—C11—C11A	−176.06 (11)	C11A—C11—O17—C17	−155.86 (10)
C7A—C10A—C11—C11A	−0.40 (16)		

### Hydrogen-bond geometry (Å, º)

**Table:**

*D*—H···*A*	*D*—H	H···*A*	*D*···*A*	*D*—H···*A*
C3—H3···O13^i^	0.95	2.44	3.2840 (14)	147
C9—H9*A*···O17^ii^	0.99	2.46	3.2207 (15)	133

Symmetry codes: (i) −*x*+1, −*y*+1, −*z*+1; (ii) −*x*+1, −*y*, −*z*+1.
